# The acrocentric part of der(Y)t(Y;acro)(q12;p1?2) contains D15Z1 sequences in the majority of cases

**DOI:** 10.1038/s41439-021-00163-9

**Published:** 2021-07-28

**Authors:** Sigrid Fuchs, Jasmin Lisfeld, Stefanie Kankel, Luisa Person, Thomas Liehr

**Affiliations:** 1 Institute of Human Genetics, University Medical Center Hamburg, Hamburg, Germany; 2 Jena University Hospital, Friedrich Schiller University, Institute of Human Genetics, Jena, Germany

**Keywords:** Genetics research, Genetic counselling

## Abstract

Chromosomal heteromorphisms (CHMs) are currently largely disregarded in human genetic diagnostics. One exception is der(Y)t(Y;acro)(q12;p1?2), which has at least been mentioned in karyotypes and discussed in reports. This derivative is frequently observed in healthy males with idiopathic infertility, which is not uncommon for CHMs. Here, we present the first systematic fluorescence in situ hybridization (FISH)-based study of 7 carriers of der(Y)t(Y;acro)(q12;p1?2). Specific probes for 15p11.2 (D15Z1) and 22p11.2 (D22Z4) were applied to answer the question of whether either of the short arms may be involved in the formation of the derivative Y-chromosome. In 6 out of 7 cases, specific staining was achieved using the D15Z1 probe, while the derivative acrocentric chromosomal region was not positive for D22Z4 in any of the 7 cases.

In conclusion, this study implies that the acrocentric chromosomal region is derived from chromosome 15 in the majority of cases with der(Y)t(Y;acro)(q12;p1?2).

## Infertility: The origin of a Y-chromosome abnormality

Researchers in Germany have clarified the origin of an abnormality in the Y-chromosome linked with infertility. Some chromosome regions are susceptible to variation, such as translocation with segments of other chromosomes. A team of researchers led by Thomas Liehr of the Jena University Hospital, investigated a Y-chromosome abnormality in seven men. Visual analysis of the chromosomes of all seven showed an aberration at the tip of the long arm of the chromosome, probably derived from an exchange with another chromosome. The researchers then used fluorescent probes to characterize the Y-chromosome. A general probe that binds the short arm of any acrocentric chromosome (chrs. 13, 14, 15, 21, 22) generated a signal. Further testing with probes for specific chromosomes revealed that the aberrant region is derived predominantly from the short arm of chromosome 15.

## Introduction

In routine human banding cytogenetics, specific chromosomal regions are prone to showing variation. In addition to the pericentric regions of chromosomes 1, 9, and 16, the long arm of the Y-chromosome and, especially, the short arms of all acrocentric chromosomes are most frequently affected by changes in size and structure. These changes that are not associated with adverse clinical outcomes and are passed down through many generations are called chromosomal heteromorphisms (CHMs)^1,2^. The new formation of CHMs has rarely been identified^3,4^.

In a study conducted in the general population, shortened acrocentric short arms were found in 34/30,117 individuals (0.11%) and enlarged acrocentric short arms were found in 149/39,773 individuals (2.38%)^1^. As all five human acrocentric chromosomes (i.e., 13, 14, 15, 21, and 22) have short arms that contain (if at all) only a few genes, size variations in these regions have no measurable influence on the carrier’s phenotype. The only known genetic contribution of the short arms is that all of them typically carry the nucleolus organizing region (NOR) in their p12 band, with approximately 40 copies on average, for a total of 300–400 copies per cell^5^.

Size variation among acrocentric short arms may occur for several reasons. There can be amplification or loss of material from each of their bands or subbands, which are referred to as p11.2, p12, and p13 for all acrocentric chromosomes. Meiotic translocations due to unequal crossover were suggested as one possible reason for p-arm variations by Malcolm Fergusen-Smith in 1974^6^, which was proven in 2006 in one case with de novo der(21)t(Y;21)(q12;p13)^3^. With the development of molecular cytogenetic technology and the availability of probes for 15p11.2 (D15Z1) and 22p11.2 (D22Z4), derivatives such as der(13)t(13;15)(p11.1;p11.1), der(21)t(15;21)(p11.1;p11.1) and der(21)t(21;22)(p11.1;p11.1) have been reported^2^.

In a subset of the 2.38% individuals in the abovementioned population with short arm enlargement of an acrocentric chromosome, this situation is due to der(acro)t(Y;acro)(q12;p1?2). As reported in 1979^7^, chromosome 15 is the most frequently involved chromosome (52%), followed by chromosomes 22 (33%), 21 (7%), 13 (4%), and 14 (4%). We suggest that most, if not all, of these acrocentric derivatives represent only one side of the coin, i.e., balanced rearrangements originating in single ancestors that might have segregated independently in two unbalanced forms with no major adverse effects on their carriers and spread through the human population. It was suggested in 1993 that the closest homology of 15p and 22p is shared with Yq12, which could explain why XY bivalents are frequently seen in close proximity to NORs during prophase of male meiosis^8^. Derivatives der(Y)t(Y;acro)(q12;p1?2) are repeatedly seen in human cytogenetic findings; some carriers are found by chance in prenatal or parental studies, and others are found among infertile males or children with congenital abnormalities. Similar observations were reported for CHMs of the pericentric region of chromosome 9^4^. However, no study has determined the frequency at which individual acrocentric chromosomes are involved in this chromosomal rearrangement. Only two exploratory orks are available, which found der(Y)t(Y;15)(q12;p11.2) to be involved in 5/8 cases^4,9^.

As chromosomes 15 and 22 are the most frequently identified acrocentrics related to der(acro)t(Y;acro)(q12;p1?2) and probes are available for 15p11.2 and 22p11.2, we performed the first systematic study of carriers of der(Y)t(Y;acro)(q12;p1?2).

**Table 1:** Studied cases, few clinical details and result are summarized here.

case	gender	age	diagnoses	result
1	Male	31 years	Infertile	46,X,der(Y)t(Y;15)(q12;p11.2)
2	Male	prenatal	Advanced maternal age	46,X,der(Y)t(Y;15)(q12;p11.2)
3	Male	37 years	Infertile	46,X,der(Y)t(Y;15)(q12;p11.2)
4	Male	36 years	Infertile	46,X,der(Y)t(Y;15)(q12;p11.2)
5	Male	3 days	Hexadactyly polycystic kidneys^a^	46,X,der(Y)t(Y;15)(q12;p11.2)pat
6	Male	10 years	Mental retardation, autism; father not available for studies	46,X,der(Y)t(Y;15)(q12;p11.2)
7	Male	40 years	Normal male	46,der(Y)t(Y;acro)(q12;p1?2)

^a^Underlying genetic cause detected by exome sequencing.

## Material and methods

Chromosome spreads were produced according to standard procedures from PHA-stimulated cultured lymphocytes or the amnion cells of seven male individuals with different clinical indications (Table 1); one male with a normal Y-chromosome was included as a control^10^. Karyotyping was performed based on GTG-banding^10^. Fluorescence in situ hybridization (FISH) was performed as previously reported^10^ using the following probes: commercially available Yq12 (DYZ1) probe, 15p11.2 (D15Z1) probe, subtelomeric Xq/Yqter probe (Abbott/Vysis, Wiesbaden, Germany), 22p11.2 (D22Z4) probe^11^, and a probe for all acrocentric short arms (midi54 microdissection-derived^12^ and/ or acro-p-arm probe (Cytocell, Oxford Gene Technology, Cambridge, UK) (cases 5 and 6)).

## Results

**Fig. 1:**
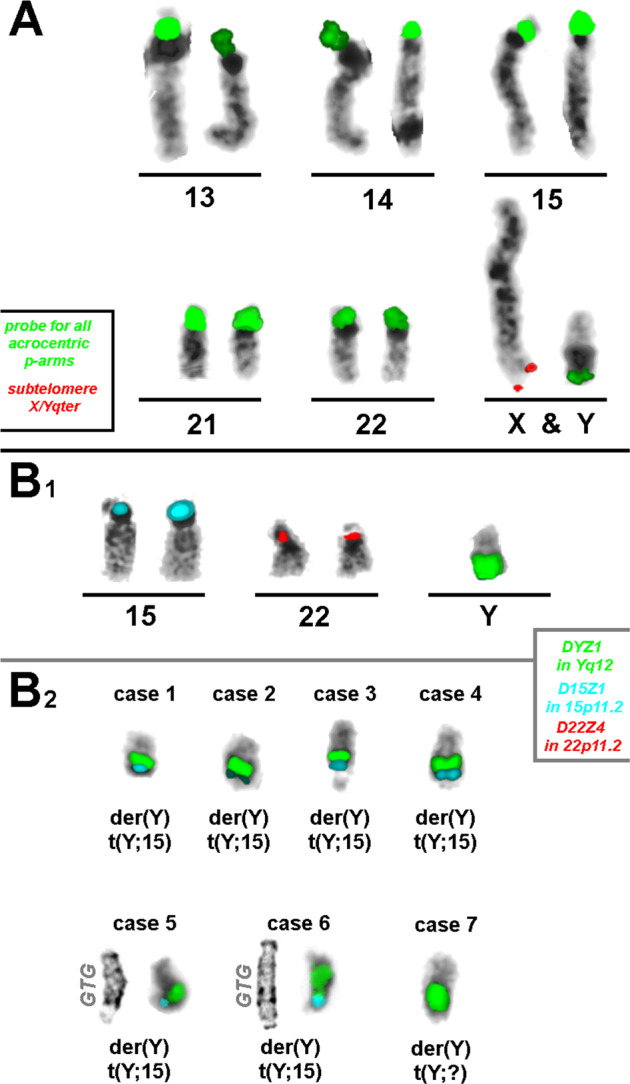
The results of fluorescence in situ hybridization (FISH) in seven individuals with der(Y)t(Y;acro)(q12;p1?2) are depicted together with the applied probe sets. The information for the label color for each probe is provided in the legends in parts A and B, including the name and location of each probe applied in the corresponding color. A probe for all acrocentric p-arms indicated that a part of this region was added in Yqter. A subtelomeric probe for Xqter/Yqter showed a signal only on the X-chromosome, indicating a loss of the tip (PAR2) from the long arm of the Y-chromosome. B1) Partial karyogram of a normal male showing the expected signal pattern for hybridization with probes DYZ1 in Yq12, D15Z1 in 15p11.2, and D22Z4 in 22p11.2. B2) Results of the probe set shown in part B1 of this figure for 7 carriers of der(Y)t(Y;acro)(q12;p1?2); see also Tab. 1. For cases 5 and 6, GTG-banding results are shown beside the FISH results. D15Z1 sequences show high signal intensity in cases 1, 3, 4, and 6, while in cases 2 and 5, the signal intensities are lower. Case 7 did not show any D15Z1 signal on the der(Y). None of the 7 cases showed a D22Z4 signal on der(Y).

In all seven cases, karyotyping revealed a derivative Y-chromosome with an aberrant tip of the q-arm, appearing as satellites in most cases. All cases were further studied by FISH analysis with a probe for all acrocentric short arms (midi54 or acro-p-arm) and the subtelomeric probe for Yqter. On every der(Y) analyzed with a probe for all acrocentric short arms, a faint to strong signal was seen. However, no signal could be detected with the subtelomeric probe for Yqter (Fig. 1A). Subsequently, hybridization was carried out with the probes for Yq12, 15p11.2, and 22p11.2 (Fig. 1B_1_). In six of the seven cases, the additional material on the long arm of the Y-chromosome showed signals of the D15Z1 probe. The signals were strong in 4/6 cases and weak in 2/6 cases. The seventh case showed no signal for either D15Z1 or D22Z1 (Fig. 1B_2_).

## Discussion

Both the der(acro)t(Y;acro)(q12;p1?2) and der(Y)t(Y;acro)(q12;p1?2) CHMs have been reported in the literature. It was demonstrated only for the acrocentric derivatives that chromosomes 15 and 22 were involved in 52% and 33% of cases, respectively^7^, while there are limited corresponding data for the reciprocal der(Y)^4,9^. This is surprising, as (i) D15Z1 and D22Z4 probes have been available for FISH studies for decades, and (ii) in North America, a founder effect has been traced based on a large family with der(Y)t(Y;acro)(q12;p1?2)^13^.

The results of the present study together with those of Wilkinson and Crolla^4^ and Kühl et al.^9^ support the hypothesis that der(Y)t(Y;acro)(q12;p1?2) and der(acro)t(Y;acro)(q12;p1?2) are reciprocal products of the same kind of rare event in the human population. Chromosome 15 (more precisely, D15Z1) is also the sequence most frequently involved in der(Y)t(Y;acro)(q12;p1?2), as it is found in der(acro)t(Y;acro)(q12;p1?2)^7^. It is important to note that it could be proven only that D15Z1 is most often involved in der(Y)t(Y;acro), as the possibility that some of the D15Z1-positive short arms could be derived from chromosome 14 (~12% of the population), 13, 21 or 22 (0.5–4% of the population) must also be considered^1^. However, based on the available data for der(13)t(Y;13), der(14)t(Y;14), der(21)t(Y;21) and der(22)t(Y;22)^7^, this seems unlikely. Therefore, we recommend that in the case of proven D15Z1 involvement, the notation der(Y)t(Y;15)(q12;p11.2) should be used.

Furthermore, these molecular cytogenetic data enabled us to distinguish at least two similar events in the human population: t(Y;15)(q12;p11.2-distal) and t(Y;15)(q12;p11.2-proximal), leading to der(Y) with a less or more intense D15Z1 signal, respectively. According to the literature^14^, it is still challenging for second- and third-generation sequencing approaches to access repetitive DNA stretches of several megabase pairs in length; thus, the determination of the ancestral founder origin and/or recurrent translocation by unequal meiotic crossing over for t(Y;15)(q12;p11.2) may only be possible in specialized labs^15^. der(Y)(Y;15) is not suspected of having any apparent effect on the phenotype of the carrier in any of the examined cases.

In conclusion, we provide the first evidence that the derivatives der(Y)t(Y;acro)(q12;p1?2) and der(acro)t(Y;acro)(q12;p1?2) observed in the general population are indeed two sides of the same coin due to the translocation t(Y;15)(q12;p11.2). In our small study group of 7 patients, we demonstrated that this event occurred at least twice.

## Disclosure

The authors did not receive any support for this work.
